# Progressive Biocatalysts for the Treatment of Aqueous Systems Containing Pharmaceutical Pollutants

**DOI:** 10.3390/life13030841

**Published:** 2023-03-20

**Authors:** Elena Efremenko, Nikolay Stepanov, Olga Senko, Olga Maslova, Ilya Lyagin, Aysel Aslanli

**Affiliations:** Faculty of Chemistry, Lomonosov Moscow State University, Lenin Hills 1/3, 119991 Moscow, Russia

**Keywords:** pharmaceutic pollutants, enzymes, bacteria, fungi, microalgae, microbial consortia, immobilized biocatalysts, biodegradation, wastewater, removing efficiency

## Abstract

The review focuses on the appearance of various pharmaceutical pollutants in various water sources, which dictates the need to use various methods for effective purification and biodegradation of the compounds. The use of various biological catalysts (enzymes and cells) is discussed as one of the progressive approaches to solving problems in this area. Antibiotics, hormones, pharmaceuticals containing halogen, nonsteroidal anti-inflammatory drugs, analgesics and antiepileptic drugs are among the substrates for the biocatalysts in water purification processes that can be carried out. The use of enzymes in soluble and immobilized forms as effective biocatalysts for the biodegradation of various pharmaceutical compounds (PCPs) has been analyzed. Various living cells (bacteria, fungi, microalgae) taken as separate cultures or components of natural or artificial consortia can be involved in biocatalytic processes under aerobic or anaerobic conditions. Cells as biocatalysts introduced into water treatment systems in suspended or immobilized form are used for deep biodegradation of PCPs. The potential of combinations of biocatalysts with physical–chemical methods of wastewater treatment is evaluated in relation to the effective removing of PCPs. The review analyzes recent results and the main current trends in the development of biocatalytic approaches to biodegradation of PCPs, the pros and cons of the processes and the biocatalysts used.

## 1. Introduction

Both the growth of the world’s population and the associated active use of various pharmaceutical compounds (PCPs) (antibiotics, hormones, cardiovascular, analgesics, anticonvulsants, anti-inflammatory and antiepileptic drugs, etc.) have led to the problem of their contamination of water and the environment [1]. Many of them can have an adverse effect on human health and are able to form a negative response to the effect they cause, which can be cumulative [2]. PCPs are found in various concentrations in the wastewater of urban sewage treatment plants, while the presence of mixtures of pharmaceutical micropollutants is noted [3,4,5,6]. Sewage treatment plants are the main facilities where PCPs with wastewater from municipal and industrial places enter and where they should be removed. The physical–chemical processes of their removal using membrane filtration, chlorination, ozonation and photocatalytic oxidation, sorption and microbiological degradation ensure the elimination of these pollutants, but have certain limitations in the effectiveness of these processes [3,4,5]. As a result, these substances often “slip through” treatment facilities and enter further into natural water sources (groundwater, rivers, seas), and from there they enter drinking water [4,5,6].

The best results in the removal of pharmaceuticals from wastewater (purification efficiency up to 90%) were achieved using ultrafiltration [7] followed by additional adsorption on a carbon filter [8]. However, the low adsorption capacity, low selectivity, high cost and long duration of the process limit the use of such methods of wastewater treatment with PCPs. In addition, sorbents are ineffective against nanoconcentrations of PCPs [8]. Disposal or recycling of used adsorbents contaminated with PCPs also causes a significant problem. Other technologies for the removal of PCPs using active oxygen forms generated by different methods [9,10], with rare exceptions, are slightly less effective, ensuring the removal of substances at an average of 65–88%. They are characterized by significant energy consumption and the formation of toxic by-products (free radicals, oxidized derivatives of pharmaceutical pollutants and the products of their destruction).

The development of membrane filtration systems makes it possible to achieve high efficiency in the removal of pollutants. For example, the initial concentrations of PCPs after primary treatment can be reduced by 10 times in wastewater [11]. However, the use of membranes is severely limited due to the high cost of their operation and maintenance. Hence, there is a need to combine membrane filtration with other methods of wastewater treatment to remove PCPs from them. Here, enzymes that can be immobilized on membrane materials are considered as a candidate for such a combination. 

Thus, there is a need for the use of biological variants for the cleaning of wastewater from PCPs that can reduce concentrations of micropollutants to an undetectable level [12,13]. In this regard, there is increasing interest in the development and use of biological methods of wastewater treatment for PCPs elimination. Biological treatment has a number of significant advantages that consist in the use of natural biocatalysts (enzymes, microorganisms and their consortia) for the destruction of micropollutants [14,15,16,17]. Therefore, the natural potential of such biocatalysts is actually used, and the processes with their participation are “nature-like”. They make it possible to obtain biodegradable products of destruction of PCPs with reduced toxicity to environmental objects, or even to completely remove the pollutants and their decomposition products from sewage via thorough biodegradation.

This review aimed to analyze the current state of developments in the field of biological purification of aquatic media contaminated with various pharmaceutical pollutants. This analysis was focused on identifying the main trends in the use of various biocatalysts for wastewater treatment from PCPs and evaluating the effectiveness of their functioning. Enzymes, bacterial cells, microscopic fungi and microalgae were considered as such biocatalysts. Particular attention was paid to the feasibility of using different cell consortia, including artificially created ones. In addition, the review aimed to compare the effectiveness of the use of biocatalysts in free and immobilized form for the degradation of different classes of PCPs and their mixtures. At the same time, the analysis used the results of studies published over the past 5 years.

## 2. Enzymatic Biocatalysts Applied for the Degradation of Pharmaceutical Pollutants

Analysis of recently published studies on the use of soluble and immobilized enzymes as biocatalysts for the biodegradation of various PCPs showed the effectiveness of these applications. Further, we decided to consider the results obtained using different forms of enzymes separately in order to highlight the main trends in recent developments.

### 2.1. Free Enzymes in the Biodegradation of PCPs

Among the soluble enzymes, the use of which has been studied in the bioprocessing of various samples of real and model wastewater contaminated with different PCPs (Table 1) [18,19,20,21,22,23,24,25,26,27,28], the most frequent use of laccase isolated from various sources, mainly of fungal origin, should be noted for these purposes. The reason for this is that laccase (EC 1.10.3.2) is one of the most studied extracellular enzymes, which can destroy a wide range of aromatic compounds. Since laccase requires only oxygen as an electron acceptor for the conversion of the substrate and has low substrate specificity, it is a universal enzyme widely used in the biodegradation of various xenobiotics.

The enzymatic degradation of various PCPs (hormones, antibiotics, cytostatic drugs), as well as pesticides and personal hygiene products, was investigated under the action of laccase in wastewater. Laccase was highly active against many target pollutants. However, in most successful cases, the degradation efficiency was 95–98% [21,24,25,26,27]. At the same time, only in a few variants of the studied media did the concentration of PCPs decrease by 100% for 16–24 h [16,23,24]. This process was particularly effective when using genetically improved variants of mutant laccase [26].

The significantly high initial rates of enzymatic degradation of many micropollutants should be noted [20]. It has been shown that the components of wastewater (micropollutants and solid suspended microparticles) can greatly reduce the activity of the enzyme, acting as inhibitors and sorbents. The loss of enzymatic activity can reach 66% [19].

The effectiveness of pollutant degradation depends on the chemical structure of the target compounds and the pH of the treated medium. For instance, a high percentage of degradation (82.4–86.3%) of bisphenol A, 2-hydroxybiphenyl and 4-tert-butylphenol was found, which was explained by the presence of a hydroxyl group in the aromatic structure of these compounds [19]. The conducted assessment of the toxicity of wastewater after its enzymatic treatment using soluble laccase showed a clear decrease in ecotoxicity [19,20]. The degradation of anticancer drugs, such as doxorubicin under the action of laccase was investigated. The highest enzymatic activity was achieved at 30 °C and pH 7, which corresponded to the characteristics of wastewater treatment plants [20].

Carbamazepine (an anticonvulsant and mood-stabilizing drug used to treat epilepsy and bipolar disorder) is one of the PCPs most commonly detected in wastewater, and has adverse effects on human and animal health [21]. Oxidation is an effective method of carbamazepine removal, but it has a number of disadvantages, such as the need for continuous supply of O_3_ or H_2_O_2_ and subsequent removal of toxic oxidation catalysts, such as active radicals. Biocatalytic degradation of carbamazepine using laccase in the presence of redox mediators is a promising approach to its removal from wastewater.

Under optimal conditions of biotransformation, with the use of laccase (35 °C, pH 6.0) and the addition of a mediator (2′-azino-bis (3-ethylbenzothiazoline-6-sulfonic acid) to the medium, the efficiency of the process reached 95%. Moreover, the resulting products of the enzymatic degradation of carbamazepine did not have the effect of estrogenicity [21]. In fact, the removal efficiency of the difficult-to-decompose carbamazepine at water treatment plants is below 20%, which leads to its detection in aquatic environments, including ground and even drinking water. This problem is due to the fact that this substance contains a strong electron acceptor amide group in its structure. When using laccase to influence carbamazepine in various investigations [16,21,24], different degradation results were obtained (from 20% to 95%). The adsorption of this substance on the inner surface of the applied bioreactors turned out to be one of the reasons that worsened its bioavailability for the effects of laccase.

When analyzing the identified possibilities of using fungal laccase for the biodegradation of various PCPs, a number of interesting results were noted. For example, laccase ensured the oxidation of 17ß-estradiol both in natural water and in the complex environment of pig manure [22]. At the same time, this biodegradation efficiency was higher than 91%, while this process took place at pH 5.0, which is characteristic of pig manure due to the increased concentration of organic acids in it. The significance of this result should be noted, since the removal of estrogens during wastewater treatment makes it possible to reduce the number of highly toxic pollutants that cause metabolic disorders and even carcinogenic risks in animals and humans.

Laccase proved to be effective in the biodegradation of a widely used heat-resistant antibiotic, chloramphenicol, in the composition of wastewater [23]. At the same time, as a result of the enzymatic reaction, chloramphenicol aldehyde was formed, which did not show toxicity to a number of bacterial and yeast cells, whereas non-enzymatic acid-base catalysis and hydrolysis of the antibiotic by the amide bond led to dehalogenation and formation of a large number of toxic compounds.

There are a number of PCPs that are regularly found in wastewater, groundwater and drinking water: diclofenac, trimethoprim, carbamazepine and sulfamethoxazole. This is due to their inefficient degradation at wastewater treatment plants using oxidation methods (ozonation, UV photolysis and UV/H_2_O_2_) [24]. Therefore, the degradation of these compounds under the action of laccase was investigated. It emerged that the biodegradation of these substances individually was more effective than in mixtures under the same conditions created for the enzyme. The obtained products of enzymatic treatment of each pharmaceutical pollutant were recognized as non-toxic [24].

Sulfamethoxazole is an antibiotic with a wide spectrum of antimicrobial action, used for bacterial infections of the urinary tract, bronchitis and prostatitis. This substance is also difficult to decompose and is often found in wastewater. It has been shown that under the action of laccase, it is possible to achieve a fairly successful removal of this substance from wastewater [24,26]. The most effective degradation of sulfamethoxazole was observed in a mixture with acetaminophen, which acts as a mediator for biocatalysis. This result suggests that the decomposition of sulfamethoxazole may be enhanced in the presence of higher concentrations of acetaminophen [16].

It has been shown that the activity of fungal laccase when exposed to triclosan clearly increases in the presence of Cu^2+^ ions in wastewater, which act as a co-factor for this enzyme [25]. Compared with the reaction without Cu^2+^ (67.17%), the efficiency of triclosan degradation increased to 95% in 4 h in the presence of 3.0 mM Cu^2+^. The analysis of inhibition of the growth of freshwater microalgae (*Chlamydomonas reinhardtii* and *Scenedesmus obliquus*) showed that the products of the enzymatic reaction showed less toxicity toward the microalgae than the original pollutant.

It should be noted that not only laccase but also peroxidase was used to degrade different PCPs: triclosan, sulfamethoxazole and steroids (estrone, 17β-estradiol, 17α-ethynylestradiol) [27,28]. It was found that peroxidase can efficiently decompose (≥95%) all pollutants, with the exception of sulfamethoxazole at a neutral pH value for 3 h in the presence of H_2_O_2_ [27].

Thus, PCPs can be degraded by enzymes such as laccases and peroxidases. However, a number of problems limit their use; in particular, low laccase activity at neutral pH values or the need to add H_2_O_2_ as a substrate for peroxidase in wastewater [19]. In this case, preference was given to laccase for use in biodegradation of PCPs, but stabilization of this enzyme was required to ensure its effective and long-term functioning, especially in the case of varying the pH of the treated media. In addition, there is a need for cheap enzymes that can be consumed in large quantities to process wastewater or to obtain biosystems with the possibility of their reuse. The immobilization of the enzymes is oriented toward the overcoming of these problems.

### 2.2. Immobilized Enzymes in the Biodegradation of PCPs

According to the information performed in Table 1, biotransformation of organic compounds using enzymatic biocatalysts is an environmentally attractive addition to traditional wastewater treatment. However, the loss of activity by enzymes in the process of their use is a serious problem. In this regard, the use of immobilized forms of enzymes capable of catalyzing the degradation of various PCPs was studied (Table 2) [8,11,29,30,31,32,33,34,35,36,37,38,39,40,41,42,43,44,45,46].

One of the promising methods of immobilizing enzymes, in particular laccases, is the production of cross-linked enzyme aggregates (CLEAs). This method of enzyme immobilization is well-known and has already proven itself positively, including with regard to laccase for municipal wastewater purification [31]. It consists in binding the amino acid residues of enzymes to each other using a crosslinking agent, and allows the maintenance of high (up to 70%) enzyme activity. CLEAs can be obtained on the basis of pure enzymes and crude proteins, which is a significant advantage in obtaining enzymatic biocatalysts for PCPs degradation in large-scale processes. However, there are a number of problems associated with the low mechanical stability of CLEAs and their fragility, which complicates mass transfer, limits the filterability of media and limits their use in industry [18,31].

An effective solution was to attach laccase to chitosan by forming an imine group between the aldehyde groups of the enzyme and the amine groups of chitosan, and then silanization of the polymer structure was carried out by adding (3-aminopropyl) triethoxysilane (APTES) [18]. Such chemical immobilization of the laccase made it possible to obtain cross-linked aggregates of the enzyme, which maintained stable catalytic activity in the pH range 6–9 and the temperature range 4–60 °C, as well as in the presence of salts (CaCl_2_, ZnCl_2_), methanol, ethylene diamine tetraacetic acid and components of municipal wastewater. After 5 oxidative cycles of PCPs, the stabilized laccase retained 67% of its initial activity.

Graphene materials are known to increase the rate of chemical reactions by improving the kinetics of electron transfer. Due to a highly stable two-dimensional layered structure, large surface area and pore volume, graphene materials were considered as promising carriers for immobilized enzymes, enabling the performance of various practical tasks, including wastewater purification. Pristine graphene is considered to be the most environmentally acceptable in the family of graphene materials. The immobilization of enzymes on this carrier was carried out by the non-covalent π-π stacking and hydrophobic interactions. It was found that the presence of pristine graphene significantly improved the removal of labetalol (β-blocker) from the medium using 2,2′-azinobis-(3-ethylbenzothiazoline-6-sulfonate) (ABTS) as a mediator. ABTS can be oxidized by laccase to form a stable cation radical. Since labetalol is not a direct substrate of laccase, the formation of an ABTS cation radical with a high oxidation potential can lead to the transformation of labetalol [29]. The enzyme immobilized on pristine graphene retained up to 60% activity in the pH range 2–10, had increased thermal stability and lost only 10% activity for 40 min at 70 °C. The efficiency of the available labetalol was 100% for 10 working cycles of the immobilized enzyme, and then it decreased by 30% during the two subsequent working cycles of the immobilized laccase. With all the advantages of this biocatalyst, it was found that 6% of labetalol from the medium was sorbed on graphene, which is an obvious drawback in this biocatalytic system.

Discussing the prospects for the use of immobilized enzymatic biocatalysts for wastewater treatment from PCPs, emphasis should be placed on the need to use carriers for enzyme immobilization that can be prepared from affordable and cheap materials with high chemical and thermal stability that are capable of functionalization and ensuring possible reuse. For these purposes, the implementation of synthetic polymers has certain advantages, since such materials have many functional (carbonyl, carboxyl, hydroxyl, epoxy, amine and alkyl) groups that provide functionalization of the polymer surface and effective binding of the enzyme. In this regard, interesting results and rather high levels of biodegradation of various PCPs were obtained using laminated nanofibers from poly(acrylic acid) [32], polyimide aerogels [33], nanofibers from poly(L-lactic acid)-co-poly(ε-caprolactone) (PLCL) [34], poly(vinylidene fluoride) membranes [37,41], polyamide granules activated by branched polyethylenimine [42] and polypropylene beads [43] as synthetic carriers for immobilized laccase.

It is known that magnetic nanoparticles can also be functionalized to immobilize laccase [35]. The experiments performed to remove diclofenac using such an immobilized enzyme were 20% higher than that of soluble laccase; however, this biocatalyst lost activity relatively quickly, and on the fourth working cycle, the biodegradation of this nonsteroidal anti-inflammatory drug was only 19%. Interestingly, when using carriers derived from natural sources, particularly biochar from coniferous wood, for the immobilization of laccase, the enzyme retained 40% activity after five cycles of diclofenac treatment. [39]. It should be emphasized that interest in carriers based on natural materials (bentonites [38], silica and activated carbons, including bio-carbons, which are obtained by pyrolysis of biomass [40]) continues to be high. Covalent immobilization on these laccase carriers makes it possible to obtain fairly active biocatalysts, and at the same time there are no restrictions on the possible obtaining of such biocatalysts on a large scale for wastewater purification from antibiotics.

To increase the efficiency of PCP removal, researchers are trying to combine physical–chemical methods of wastewater treatment with the use of immobilized laccase. In this regard, an interesting solution is the combination of electrooxidation (EO) with enzymatic treatment of wastewater. During electro-oxidation, highly oxidizing hydroxyl radicals are generated using special electrodes. By itself, EO is ineffective for removing PCPs that are present in low concentrations in wastewater. At the same time, high energy costs are required to remove PCPs by this method from the total volume of treated wastewater. A study of the purification of municipal wastewater from triclosan using electrochemical processes in combination with treatment by laccase immobilized on TiO_2_ nanoparticles showed that the degradation efficiency of triclosan reached 93% [30]. The TiO_2_ nanoparticles used for such immobilization are characterized by high chemical stability and simplicity of functionalization, for which crosslinking agents can be applied [36]. The laccases thus immobilized showed high thermal stability at 50 °C (the half-inactivation period was 45.7 h) and high stability at low pH values of 2 and 3 (the half-inactivation period was 31.8 and 107.1 h, respectively). However, a decrease in the activity of the immobilized enzyme was revealed due to the appearance of F^−^, Cl^−^ or Br^−^ anions in water, as well as nitrite and cyanide, blocking the access of substrates to the active center of laccase [36].

In another study, membrane filtration was combined with enzymatic treatment of wastewater [11]. Phenol oxidases (laccase and tyrosinase) were used in this combined process. It is known that tyrosinase forms o-diphenol from the initial compound and subsequently releases oxidized, usually highly reactive o-quinone, which slowly polymerizes as a result of auto-oxidative processes. Laccases oxidize phenolic compounds with the formation of corresponding free radicals, which lead to the formation of molecules prone to polymerization. As a result of polymerization, macromolecules are formed, which are easier to remove from the treated solution. The efficiency of degradation of 14 PCPs from urban wastewater was studied using microfiltration polysulfone hollow fiber membrane with phenol oxidases immobilized on it [11]. As a result, high efficiency of removal (90%) of anti-inflammatory drugs (acetaminophen, naproxen, mefenamic acid, ibuprofen, ketoprofen, indomethacin) from sewage was achieved within 24 h. Removal of acetaminophen and mephenamic acid occurred three times faster (>85% for 8 h).

The use of a combination of enzymes for the degradation of atenolol ensured the high efficiency of the process (100% for 120 h), and the degradation of bezafibrate, cofein, carbamazepine and fenofibrate was 80%, 70%, 40% and 30%, respectively. When using a membrane with enzymes, complete removal of all PCPs was achieved within 5 days. At the same time, the enzymes retained up to 70% of their initial catalytic activity during the entire period of PCP treatment [11].

It should be noted that researchers today are interested not only in developing new options for the immobilization and use of laccases, but also in other enzymes. Thus, in a number of new publications on the destruction of β-lactam antibiotics in wastewater, information has appeared about the successful use of β-Lactamase [44,45]. This enzyme provided the biodegradation of antibiotics in real wastewater containing cefamesin, amoxicillin and ampicillin (50–100 mg/L) simultaneously. The efficiency of the process reached 72.3–92.8% for 20 days.

β-Lactamase covalently immobilized on Fe_3_O_4_ nanoparticles showed excellent stability and the possibility of reuse for at least 10 batch cycles to degrade penicillin (5 mg/L) for 5 min. After 30 working cycles, the degradation efficiency decreased by 95%; however, the concentration of the antibiotic used in the experiments exceeded the concentration (0.153 mg/L) by 30 times, which is usually determined in the wastewater of the pharmaceutical industry [45].

Chloroperoxidase, immobilized on dendritic silica particles and coated with an amyloid-like protein nanofilm, retained up to 80% of the initial catalytic activity during 20 cycles of enzyme use for antibiotic biodegradation. More than 80% of the levofloxacin and rifaximin present at a concentration of 100 mg/L decomposed under the action of this enzyme within 0.5 h [46].

Another trend that can be noted in studies on the enzymatic biodegradation of PCPs in wastewater is to study the possibility and feasibility of combining immobilized enzymes possessing different “non-selectivity” of action against various substrates; for example, horseradish and lignin peroxidases. This technique expands the range of substrates that can undergo bioconversion and the range of conditions for the implementation of PCPs biodegradation. In particular, the use of immobilized peroxidases in the decomposition of diclofenac, carbamazepine and paracetamol was investigated, and it was shown that horseradish and lignin peroxidases immobilized on Fe_3_O_4_ nanoparticles and encapsulated in SiO_2_-sol-gel retained 43–50% of their activity at 55 °C after 20 consecutive operating cycles for 24 h. A decrease in pH to 3.0 significantly increased enzyme activity, and the efficiency of the degradation of diclofenac, carbamazepine and paracetamol by both peroxidases reached 100%, 100% and 50%, respectively, within 72 h. Meanwhile, the use of only one lignin peroxidase or horseradish peroxidase at pH 5.0 ensured 59%, 60%, 9% and 64%, 68% and 9% destruction of the same PCPs [8].

Thus, the use of enzymes, including in immobilized form, in combination with other enzymes and physical–chemical methods of wastewater processing constitute one of the current directions in the development of effective approaches to the biodegradation of various PCPs in wastewater.

## 3. Bacterial and Fungal Biocatalysts for the Biodegradation of PCPs

Despite the attractiveness of enzymatic biodegradation of PCPs, the problems of the appearance of products of enzymatic reactions in wastewater remain. In this regard, there is a need to purify such wastewater with the help of aerobic or anaerobic sludge, but these processes turn out to be lengthy and not always effective. In this connection, there is a need to search for microbial cells capable not only of catalyzing the destruction of PCPs using their own enzymes, but also of utilizing the products of the destruction of these pollutants (Table 3) [47,48,49,50,51,52,53,54,55,56,57,58,59]. Degradation of various PCPs can be carried out by bacterial cells due to their activity in oxidation, reduction and hydrolysis of the PCPs and can be accompanied by primary sorption or precipitation under the influence of substances secreted by the same cells into the treated media.

Since it was found that sulfate-reducing bacteria are involved in the degradation of sulfamethoxazole under environmental conditions, the decomposition of sulfamethoxine and sulfamethoxazole by *Bacillus subtilis* AQ03 cells, which were isolated from sewage sludge, was investigated. A bioreactor with these cells was used to degrade antibiotics present in samples of real wastewater (500 mg/L). After 10 days of their cultivation, the degradation of antibiotics was 100%. It emerged that the isolated strain was characterized by maximum antibiotic resistance (>2000 ppm) and produced various enzymes: proteases, amylases, cellulases and laccases. The achieved degradation of PCPs was a consequence of the manifestation of multiple enzymatic activities by bacterial cells [47]. It was revealed that the introduction of glucose into the medium with cells as a carbon source supported their viability and improved the biodegradation of antibiotics.

It should be noted that laccase-producing cells often attract the attention of researchers as biocatalysts for the destruction of various PCPs [47,48,49,52,53,55]. At the same time, both natural strains of microorganisms isolated from sources of PCP contamination [47,48] and genetically modified cells with an increased level of laccase synthesis were studied [49]. Despite the high efficiency of the functioning of recombinant cells, their use will mostly likely be limited, since it becomes necessary to constantly introduce inducers of the synthesis of recombinant enzymes and antibiotics into environments contaminated with PCPs to maintain selective conditions for the cultivation of laccase producers.

The results on the use of microorganisms simultaneously synthesizing not only laccases, but also peroxidases, for the biodegradation of PCPs are very attractive from the practical point of view [54,55,56,57,58]. This is often achieved through the use of various consortia consisting of several cultures [52,53]. In particular, for an artificial consortium of fungi consisting of *Phanerochaete chrysosporium* and *Pycnoporus sanguineus*, successful degradation of various antibiotics (ciprofloxacin, norfloxacin and sulfamethoxazole) present in a mixture was shown. At the same time, fungal *P. sanguineus* cells synthesized laccase, and the fungi *P. chrysosporium* secreted various peroxidases (lignin peroxidase (LiP) and manganese peroxidase (MnP)), which required additional injection of H_2_O_2_ into the medium for the oxidation of substrates [52]. Complete degradation of all PCPs was achieved for 4 days due to the presence of a complex of oxidative enzymes of the fungal consortium. However, an increase in the biomass of fungi that degrade different PCPs to use them as their own substrates increases the viscosity of the treated media and often clogs filters installed on water purification systems. In addition, the loading of H_2_O_2_ into the medium is required for the effective catalytic functioning of peroxidases, and that is the backside of the process.

In general, interest in bacterial cultures synthesizing oxidative enzymes other than laccases and peroxidases is currently growing among investigators. In particular, it has been shown that several bacterial cultures (*Microbacterium* sp., *Acinetobacter* sp., *Achrombacter* sp., *Pseudomonas psychrophila*) are capable of decomposing the antibiotic sulfamethoxazole. The most active strain of *Acinetobacter* sp. was used to decompose sulfamethoxazole in a mixture with several PCPs at once for 10 h [50]. The successful degradation of sulfamethoxazole in the presence of tri-methoprim, triclosan and diclofenac has been shown. The reason was that the cells *of Acinetobacter* sp. decomposed only sulfamethoxazole by attacking its amine group in the benzene ring, which was absent in the molecule structures of trimethoprim, triclosan and diclofenac. The selectivity of the action of the bacterial cells against sulfamethoxazole left the other compounds without biodegradation under these conditions of wastewater treatment.

Diclofenac is a widely prescribed nonsteroidal anti-inflammatory drug that is currently ubiquitous in the aquatic environment. It is known that the bacterial strain *Labrys portucalensis* F11 is capable of decomposing diclofenac for its use as a single carbon source [51]. The metabolic degradation of diclofenac by the *L. portucalensis* F11 cells occurred mainly due to hydroxylation reactions under the action of the oxidative enzyme cytochrome P450.

With all the successful examples of the use of suspension bacterial and fungal cells for the destruction of various PCPs, it should be emphasized that most of them require quite a long time for realization (Table 3). To preserve the viability of cells during this long period of time, an additional carbon source should be introduced into the treated wastewater. To accelerate the processes of biodegradation, it is possible to use the high concentration of cells and keep them in this state due to immobilization. In addition, it is possible to combine them with other types of microorganisms capable of forming stable consortia and showing a synergistic effect in the biodegradation of PCPs. Further, these trends of developments of actual solutions for cleaning wastewater from PCPs are discussed in particular.

## 4. Immobilized Bacterial and Fungal Cells as Biocatalysts for Biodegradation of PCPs

In recent years, immobilized bacterial cells have been widely used for various biotechnological purposes, including those that are preferred for the biodegradation of various PCPs (Table 4) [59,60,61,62,63,64]. An analysis of the current publications (Table 4) allows us to conclude that the methods of cell inclusion in gel matrices [59] and sorption of cells on various carriers are actively used as main methods for the immobilization of bacteria [60,61,62,63,64]. Both Gram-positive and Gram-negative bacterial cells are immobilized by these methods. The duration of the use of immobilized cells for the processing of synthetic or real wastewater, as a rule, was predetermined by the complexity of degradation of the removed PCPs and the chemical nature of their molecules. The removal efficiency of pollutants was usually 90% or more. The greatest interest in the use of bacterial cells as biocatalysts for the biodegradation of PCPs can be noted in relation to bacilli [59,60] and pseudomonades [62,63,64].

The study of not only bacterial but also fungal cells in immobilized form was carried out in the processes of PCP biodegradation (Table 4) [65,66,67]. Almost all individually applied (not as a part of consortia) fungi are presented by the *Trihoderma versicolor* cells. The reason may be that they have a variety of enzymatic activities, do not synthesize their own antibiotics and are highly toxic mycotoxins. In all cases, the cultivation of fungal mycelium of *T. versicolor* on the surface of different carriers was used [65,66,67]. It has been shown that immobilized fungi have a very wide range of PCPs that can be biodegraded as substrates with an efficiency of 60% or higher. The pollutants that are most actively decomposed by immobilized fungi include antibiotics, the degradation of which in most cases exceeds 90% (Table 4).

The high efficiency of use of *T. versicolor* fungal cells as successful biocatalysts was established for the treatment of real hospital wastewater containing 19 pharmaceutical pollutants, including antibiotics, psychiatric and anti-inflammatory drugs, β-blockers, analgesics, lipid regulators, steroid hormones, etc. During 75 days of continuous operation, the degradation efficiency of the antibiotics azithromycin, metronidazole and sulfamethoxazole reached 98.4%, 83.0% and 76.0%, respectively, in this biodegradation process [67].

One of the problems in the use of immobilized fungi is the need to ensure intensive aeration of the treated media, which is associated with significant energy costs. In this regard, microalgae are considered as a possible alternative that does not require forced aeration of the media for biodegradation removal of PCPs.

## 5. Microalgae as Efficient Biocatalysts for the Biodegradation of Pharmaceutical Pollutants 

The possibility of using various types of wastewater for the cultivation of different microalgae is well known. It allows the combination of the growth of these photosynthetic microorganisms with wastewater treatment. The interest in the implementation of such biocatalysts for degradation of PCPs is evident [68,69].

Recently, several very informative reviews have been published on the current use of microalgae in the processes of PCP biodegradation [17,68,69,70,71,72,73]. The success of the use of microalgae for the removal of pharmaceutical pollutants is observed due to the natural abilities of these cells, and is confirmed by the fact that phototrophic microorganisms are widespread in natural, including polluted, water sources, and are actively used in other wastewater treatment processes [74,75].

**Figure 1:**
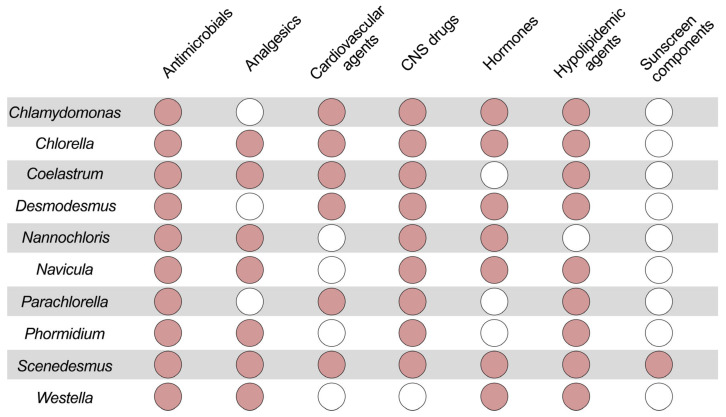
Elimination of several pharmaceuticals (antimicrobials; analgesics, including anti-inflammatory agents; cardiovascular agents; drugs for central nervous system (CNS); hormones; hypolipidemic agents; sunscreen active components) by different microalgae of genus *Chlamydomonas*, *Chlorella*, *Coelastrum*, *Desmodesmus*, *Nannochloris*, *Navicula*, *Parachlorella*, *Phormidium*, *Scenedesmus* and *Westella*. Empty circles illustrate a lack of information about eliminative activity of such microalgae towards this type of PCPs.

An analysis of the current investigations published in 2021–2023 [17,68,69,70,71,72,73] (Figure 1) showed that various microalgae cells are able to participate in the biodegradation of various PCPs. We carefully analyzed examples of the microalgae biodegradation of antimicrobials, analgesics, anti-inflammatory and cardiovascular drugs, drugs for the central nervous system (CNS), hormones, lipid-lowering agents and the active components of sunscreen. In the analysis of the possible biodegradation of these compounds, special attention was paid to those phototrophic cells (Figure 1) that were able to remove at least four types of different pharmaceutical pollutants from wastewater. Several other groups of microalgae (i.e., cells of genera *Haematococcus*, *Nannochloropsis*, *Selenastrum*, *Synechocystis* and *Tetradesmus* capable of eliminating three types of pharmaceuticals, and *Arthrospira*, *Phaeodactylum* and *Pseudonabaena*, which were capable of eliminating two types of pharmaceuticals; etc.) were discarded from Figure 1 for clarity. The results obtained clearly represent those microalgae that can be used for the biodegradation of complex PCP mixtures. It appeared that the cells of genera *Chlorella* and *Scenedesmus* were among the most “universal” biodestructors of PCPs (Figure 1).

It should be noted that in order to stabilize microalgae cells in wastewater treatment processes, they can be immobilized (Table 5) [76,77,78,79,80]. In this regard, the efficiency of the functioning of cells of the microalgae *Nannochloropsis* sp. used in a free form was compared with those immobilized in Ca-poly(vinyl alcohol) gel form in the removal of paracetamol, ibuprofen and olanzapine [76]. The results showed that free microalgae remain alive for a longer time than cells immobilized in used carriers, which indicates inhibition of cell proliferation by these polymer matrixes. However, removal of paracetamol and ibuprofen by immobilized microalgae *Nannochloropsis* sp. for one day was significantly higher as compared to the free cells.

The treatment of domestic wastewater from the hormone 17ß-estradiol under the action of microalgae *Desmodesmus* sp. WR1 cells immobilized in Ca-alginate gel was studied. The hormone removal efficiency was very high (99%). The main pathway of 17ß-estradiol metabolism by the cells was hydroxylation, O-methylation, glycosylation, dehydrogenation and dicarboxylation. The process was very successful not only owing to the good biodegradation of the pollutant, but also due to the fact that the adsorption of the hormone by Ca-alginate granules was insignificant [77].

The efficiency of carbamazepine removal by *Chlorella vulgaris* cells immobilized in the composite Ca-alginate/polyvinyl alcohol gel was quite high (95%) at a concentration of 10 mg/L of the pollutant, and decreased by only 7% with an eightfold increase in its concentration [78]. This result was significantly better than what was demonstrated by suspension cells under the same conditions. However, adsorption (10–13%) of carbamazepine by the composite carrier itself was noted.

Ceramic Al_2_O_3_ nanoparticles (0.5 g/L) were added during the immobilization of *C. vulgaris* cells in Ca-alginate gel to increase the efficiency of carbamazepine (100 mg/L) removal [79]. The efficiency of carbamazepine degradation by immobilized cells with nanoparticles was 89.6% for 4 days, which was higher in comparison with immobilized cells without nanoparticles (68.8%) and free cells of *C. vulgaris* (48.6%). Repeated working cycles for the removal of high concentrations of carbamazepine from the bio-treated medium showed the high stability of the functioning of the immobilized cells.

Thus, the immobilization of microalgae, as well as other microorganisms, leads to an increase in the efficiency of removing pharmaceutical pollutants by reducing their inhibitory effect on cells and partial adsorption on the carriers. However, despite the high efficiency (up to 99%) of biological treatment using microalgae cells, this approach is longer in duration in comparison with enzymatic treatment of wastewater. Since microalgae and cyanobacteria mainly exist in stable consortia with other microorganisms in natural water sources, many researchers focus their attention on them [80]. 

To date, it has already been demonstrated that the symbiotic interaction between microalgae and bacteria increases the efficiency of PCPs removal [81]. The use of various microbial consortia for the purification of wastewater by the “nature-like” approaches is the next step of our analysis.

## 6. Microbial Consortia for Biodegradation of PCPs

It has been established that phototrophic microorganisms are not always able to independently destroy pharmaceutical contaminants in wastewater, especially when mixtures of PCPs are present in them. Some substances are often accumulated by the biomass of phototrophic microorganisms without undergoing transformation under the action of these cells [82]. 

An analysis of recently published studies (Table 6) [81,83,84,85,86,87,88,89,90,91,92,93,94,95,96] suggests that the use of consortia of different microorganisms, including microalgae cells, can allow us to resolve the problems of biodegradation of PCPs at a higher level. In particular, the use of natural or artificial cell consortia makes it possible to expand the spectrum of degradable compounds, to carry out effective degradation of PCPs mixtures, to combine the concentration (accumulation) of PCPs and their degradation and to make this degradation thorough. 

Despite the significant number of works devoted to the use of natural biocatalysts, such as aerobic or anaerobic consortia, for the removal of pharmaceutical pollutants, the main focus of analysis of the biocatalytic processes is on the assessment of changes in the microbial composition of the consortia themselves and on biogas accumulation, whereas the destruction of specific PCPs is not controlled [97,98].

In this regard, the use of aerobic and anaerobic natural consortia for the transformation of individual PCPs is mainly reduced to their use as a basis for obtaining consortia gradually adapted to the presence of pollutants [81,83,85,86,88,89,90,99].

To reduce the time and costs of long-term adaptation of natural consortia to PCPs, it is possible to use stabilized (artificially created and immobilized) forms of microbial consortia, which are characterized by increased tolerance to the presence of various xenobiotics [94]. 

Fundamentally, an effective approach to the creation of consortia for the destruction of xenobiotics is a directed combination of cultures based on the preliminary selection of active PCPs destructors and an understanding of the possible interaction between them [80,100,101,102].

As for the conditions used to maintain the consortia of microorganisms, they do not differ from the conditions applied for the individual cultures. Usually, these are pH 6.5–8 and temperature 20–25 °C or 30–35 °C for aerobic [81,85,87] or anaerobic [83,84,86] biosystems, respectively.

In the case of degradation of pharmaceutical micro-pollutants in the composition of real wastewater, it is necessary to take into account the presence of other nutrition sources and xenobiotics. For instance, it was found that the consortium, which effectively decomposed ibuprofen as the only source of carbon, preferred to utilize a more accessible and simply degraded carbon source when glucose was added to the medium [90].

In order to give priority to the utilization of PCPs, it is necessary to introduce easily digestible substrates (glucose, acetate, glycerin, organic acids, etc.) into the media as additional sources of nutrients, not in the form of pure substances, but as a part of other waste [103]. However, such preferences of microorganisms in the presence of additional nutrition and energetic sources in the treated media still constitute the real causes of the residual amounts of different PCPs in the composition of wastewater at the outlet from the treatment facilities. This should be taken into account in the case of the development of new consortia for use in real wastewater treatment and regular monitoring of the chemical composition of the media at the entrance to the treatment facilities. The monitoring can be carried out if necessary in order to increase the retention time of the media at the stage of biotreatment, or, if possible, to increase the concentration of biocatalysts (consortia) in the bioreactor.

One of the ways to retain high concentrations of cells constituting consortia in the media with PCPs is their immobilization on different carriers [91,92,93,94,95,96]. This makes possible the processing of wastewater in periodic and continuous regimes with the extension of the ranges of applied pH values (from 4 to 9) and temperature (from 20 °C to 45 °C) [91].

It should be emphasized that it is feasible to use consortia when it is necessary to treat wastewater containing complex and multicomponent mixtures of pharmaceutical micropollutants [87,88,89].

The presence of several cultures with different substrate specificities allows them to cope more effectively with such pollutants, including their participation in the cascade processes of PCP degradation and utilization [96]. The participation of microalgae in such consortia, which require active aeration for the course of intensification of oxidative processes, reduces the load on aerating systems, but requires the organization of processes under conditions that ensure photosynthesis (usually, in open bioreactors).

## 7. Hybrid Physical–Chemical–Biocatalytic Treatments of the PCPs

Another promising approach to the problem of the necessary intensification and improvement of the efficiency of destruction of PCPs can be realized via the combination of physical–chemical methods of media treatment with the use of various biocatalysts (Table 7) [104,105,106,107,108,109,110,111,112,113,114,115,116,117].

Active sludges, microalgae, individual cell cultures, white rot fungi and enzymes were actively considered as biocatalysts for such combined-action destruction of pollutants [69,105,106,117]. The most extensive research was focused on active sludges, which contained a wide range of microorganisms with different biocatalytic activities and were actively used in existing industrial and municipal wastewater treatment systems.

The chlorination and advanced oxidation processes (AOPs) based on the use of photolysis, hydrogen peroxide, ozone, Fenton reagents, nanofiltration, sorption method and electro-chemical decomposition are the most actively studied among the physical–chemical methods used in combination with the subsequent biocatalytic treatments of aqueous media with PCPs (Table 7). These physical–chemical methods are based on the generation of AOPs, such as OH•, O_2_•− and HO_2_•, capable of decomposing various organic pollutants. Usually, hydroxyl radicals (OH•) are dominant components among AOPs. The formation of reactive particles can be triggered by UV irradiation [112], additions of oxidants [111,116] or electricity application [105,106,107,108,109,110,113,114,115]. The effectiveness of AOPs depends on the rate of formation of free radicals, as well as on the availability of contact between radicals and organic compounds.

In the process of ozonation, PCPs are under attack from radicals (O’●) formed during the decomposition of molecular ozone (O_3_), as well as hydroxyl radicals (OH●), which are formed as a result of the decomposition of ozone and its interaction with water [104]. Highly reactive hydroxyl radicals are capable of non-selective destruction of most organic and organometallic contaminants. They initiate radical chain reactions that lead to degradation resulting in the complete oxidation of organic compounds and their mineralization into CO_2_, water and inorganic ions. 

Molecular O_3_ itself is a selective electrophile that reacts rapidly with amines, phenols and double bonds in aliphatic compounds. As a result of the oxidation of organic compounds by ozone, oxygen-containing intermediates are most often formed. The short lifetime of ozone makes the processes with its use energy-intensive. In addition, when using pure ozone, the number of reactive OH● radicals with a high destructive/oxidizing potential (Eo = 3.06 V) is limited. 

As an alternative to ozone, H_2_O_2_ is considered a cheaper and more affordable but less reactive oxidizer with the potential for forming hydroxyl radicals. Fenton-type reactions have a greater oxidative reactivity compared to pure H_2_O_2_, during which, as a result of reactions between iron ions (Fe^2+^) and hydrogen peroxide, hydroxyl radicals (OH●) are formed in an acidic medium. The disadvantage of the homogeneous process is the dependence on high iron content (50–80 ppm). To increase the efficiency of the process, it is most preferable to use heterogeneous Fenton-like solid catalysts, such as [Fe(OH)_2_]^+^[Fe(H_2_O)]^+2^, [Fe(H_2_O)_6_]^+3^, Ferrous polycation, Fe_2_O_3_ and α-FeOOH.

Generation of OH• radicals can be carried out by UV irradiation of photolysis of water molecules [112]. The intermediate products of the photocatalytic action of the OH• radicals on the PCPs, such as aromatic oxides, oxides, peroxides and polyhydroxylated compounds, have high reactivity and toxicity. 

Currently, the use of heterogeneous photocatalysis is becoming increasingly widespread. The process is based on the absorption of light of a certain wavelength by a photocatalyst, resulting in electron (e−) excitations from the valence band to the conduction band of the photocatalyst (electron–hole separation). This movement generates positive holes (h+) in the valence band. Positive holes cleave molecules of H_2_O to form free radicals (OH•). Electrons (e−) in the conduction band are captured by oxygen molecules to form superoxide radicals (O_2_•−).

Among the catalysts for AOPs, various photocatalysts are known to be used, including TiO_2_, Ag/ZnO, ZnSnO_3_/ZnO/nano-ZnO, ZnS-NiS/zeolite, TiO_2_/ZnIn_2_S_4_ and Fe/graphene-TiO_2_ [114].

Due to the presence of certain limitations, the above-mentioned reagents and physical-chemical methods are rarely used individually. Most often, when implementing AOPs, one or more components from the following list are combined: bioelectrochemical systems (BES), UV, H_2_O_2_, O_3_ and Fenton reagents with or without catalysts (Table 7). At the same time, due to the expansion of the spectrum of oxidants involved in the process, a synergistic effect can be observed. For example, degradation of PCPs during photooxidation always occurs faster than during direct photolysis.

It was confirmed that the use of only physical–chemical methods to remove PCPs can lead to the destruction of pollutants with the formation of toxic products. However, in a number of cases, it has been shown that the subsequent biocatalytic treatment allows effective removal of the toxic products from the wastewater [112,118]. Thus, the combination of physical–chemical and biocatalytic methods of the destruction of micropollutants provides an optimal combination of the advantages of each method and reduces the negative effect of their disadvantages.

A comparative study showed that adsorption treatment in combination with a biological method proved to be more effective compared to ozone application and the formation of less toxic secondary intermediates from PCPs under the action of strong oxidants [104]. 

Pretreatment of pharmaceutical wastewater by Fenton reagents with subsequent treatment of the resulting medium with aerobic sludge makes it possible to quickly reduce not only the concentration of the existing PCPs, but also, in general, to significantly reduce the level of contamination with various organic compounds [116].

Today, BES in the form of microbial fuel cells (MFCs) and microbial electrolysis cells (MECs) are being developed to purify aqueous media from pollutants [113,114,115]. Their action is based on a combination of electrochemical redox reactions with biocatalytic destructive activity and microbial electrode-respiration processes. MFC uses electrochemically active microorganisms to convert chemical energy into electrical energy [119]. The main components of BES are the anode, cathode and proton exchange membrane and an external resistor, through which the formed electrons pass from the anode to the cathode. By oxidizing the substrate in the anode chamber under anaerobic conditions, biocatalysts produce electrons and protons. The electrons are transferred to the anode and then flow to the cathode. Protons simultaneously enter the cathode through the proton-exchange membrane. Electrons, protons and an electron acceptor (for example, air or oxygen) in the cathode chamber undergo a reduction reaction. In this case, a constant electric current is generated. The process attracts the attention of researchers as a potential alternative approach to solution of complex problems of decomposition of various organic pollutants, including nitro-, azo-, halogenaromatic compounds and antimicrobial agents in aqueous media [120].

The low compatibility of anodes and biofilms, expensive membranes and cathode catalysts are among the limitations of the method. Thus, the best results can be obtained via the creation and application of hybrid physical–chemical–biocatalytic treatments of the PCPs (Table 7).

An analysis of the known processes combining the use of microalgae or activated sludge with physical–chemical treatment of PCPs showed that the presence of a photocatalytic stage ensured the fastest achievement of the target results (Table 7). 

It seems that decomposition and mineralization of pollutants in BES can be further accelerated due to the presence of Fe(III) compounds in the reaction medium, which successfully integrate into a joint BES with photoactivation and activation with Fenton reagents. However, there are a lot of factors in BES-based processes that can significantly affect its effectiveness: the type of microorganisms used, electrode material, reactor architecture, pH of the medium, electrolyte concentration, substrate type, presence of electron donors and acceptors, concentration, temperature, redox properties and initial concentration of the pollutant, etc. [120,121,122,123]. Therefore, this direction needs to be explored in further scientific and practical advancements.

## 8. Comparative Analysis of Various Approaches to Biodegradation PCPs Based on Use of Enzymes and Microorganisms

**Figure 2:**
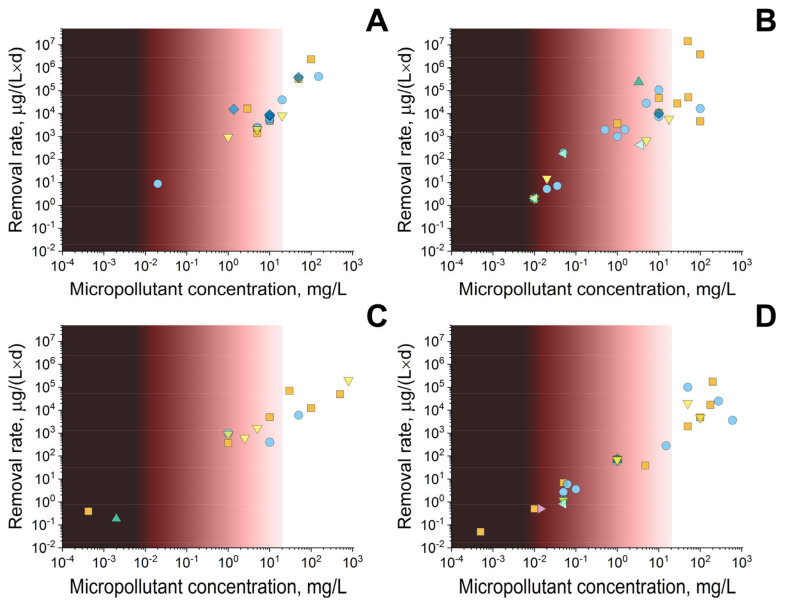
Removal rates of antimicrobials (■); analgesics, including anti-inflammatory agents (●); cardiovascular agents (▲); drugs for CNS (▼); hormones (◆); hypolipidemic agents (◀); and sunscreen active components (▶) by enzymes (**A**), immobilized enzymes (**B**), microorganisms (**C**), or immobilized microbial cells, including microbial consortia (**D**). The rates of biodegradation at different initial concentrations of PCPs were calculated on the basis of data presented in Table 1, Table 2, Table 3, Table 4, Table 5 and Table 6. The concentration range most relevant to the real conditions in effluents is indicated by the darkest color, gradually decreasing to the least relevant concentrations.

As a conclusion, we decided to collect in Figure 2 the results of our in-depth comparison and generalization of data from different authors cited in this review. Figure 2 shows the removal rates of various PCPs depending on their initial concentrations in different wastewater. 

When several compounds of the same type were studied in the cited works, only the best results of the authors were considered.

The analysis of the obtained results allowed us to draw several main conclusions:

- the range of concentrations of PCPs, the degradation of which is possible under the action of microbial cells, is wider than the equivalent indicator established for enzymes;
- the destruction rates of substances under the action of enzymes are two orders of magnitude higher than under the action of microbial cells;
- immobilized cells and enzymes in general have higher removal rates of different PCPs, as well as a wider range of these pollutants that can be biodegraded;
- enzymes, unlike microbial cells, can be successfully used for the biodegradation of hormones;
- among all PCPs, the maximum removal rates are characteristic of antimicrobial substances not only for enzymes, but also for microorganisms, which indicates the resistance of microbial biocatalysts to these pollutants;
- analgesics, anti-inflammatory agents and cardiovascular agents decompose more efficiently under the action of immobilized biocatalysts;
- it is more expedient to use microbial cells for biodegradation of drugs for CNS;
- only immobilized biocatalysts are used for the biodegradation of hypolipidemic agents, while enzymes are able to show activity in a wider range of concentrations of these substances;
- there are still few studies on the decomposition of lipid-lowering agents and active components of sunscreen.

In order to actively use enzymes, for example, laccases, in the biodegradation of PCPs, their large-scale economically and environmentally efficient production is necessary. 

The expediency of using immobilized forms of enzymes is beyond doubt, since this can increase their activity and stability, and most importantly, the possibility of their reuse. However, carriers for the immobilization of enzymes should be characterized by cheapness, the ability to retain a significant amount of enzyme per unit weight, mechanical, chemical and thermal stability and low sorption of PCPs. The same requirements apply to the carriers for immobilized cells. The use of artificial consortia in immobilized form is very promising. 

The immobilized biocatalysts showed a wider substrate range and high activity and can be used not only for the degradation of pharmaceutical pollutants but also of other pollutants like pesticides, microplastics [124], mycotoxins [125], etc.

The results of the search for new combined physical–chemical–biological approaches to the degradation of PCPs are interesting for new developments. Further, research aimed at improving hybrid technologies with increased productivity and improved environmental and economic characteristics can be expected here.

## Figures and Tables

**Table 1:** Enzymes used for degradation of pharmaceutical pollutants in various types of wastewater.

Biocatalyst [Reference]	Pollutant Concentration	Optimal Conditions of Enzymatic Action; Pollutant Degradation Efficiency
Free Enzymes		
Laccase from *Trametes hirsute* [18]	Municipal wastewater with cannabidiol (0.318 μM)	20 °C, 8 h, 135 rpm; addition of 1 mM acetaminophen as mediator; 92.0% degradation of cannabidiol
Laccases from *Trametes pubescens* MUT 2400 [19]	Samples of municipal wastewater after primary sedimentation (W1) and at the end of the process (W2) with total concentration of micropollutants equal to 403.2 μg/L and 349.5 μg/L, correspondently; bis-(2-ethylhexyl)phthalate, diethyl phthalate and ketoprofen were the most notable micropollutants in the mixtures	20 °C, pH 7.7–7.8, 24 h, 100 rpm In W1: 86.3%, 84.9% and 82.4%degradation of bisphenol A, 2-hydroxybiphenyl and 4-t-butylphenol, correspondently; up to 70% degradation of 9 micropollutants and below 50% degradation of other micropollutants; In W2: 63% and 77–81% degradation of ketoprofen and oxybenzone, correspondently
Laccase from *T. versicolor* [20]	Phosphate-citrate buffer with doxorubicin (0.25–10 mg/L)	30 °C, pH 7.0, 24 h, V_max_ = 703 µg/h/L 41.4% degradation of doxorubicin (1 mg/L)
Laccase from *T. versicolor* [21]	Buffer with carbamazepine (1 mg/L)	35 °C, pH 6.0, 24 h 95.0% degradation of carbamazepine
Laccase from *T. hirsuta* [22]	17β-estradiol in natural water (5 μmol/L) and pig manure (200 μg/kg)	25 °C, pH 5.0 94.4% and 91.0% degradation of 17β-estradiol in water (for 2 h) and pig manure (for 7 days), correspondently
Laccase from *T. hirsute* [23]	0.1 M acetate buffer with chloramphenicol (10 mg/L)	28 °C, pH 5.0, 48 h 100% degradation of chloramphenicol
Laccase from *Bjerkandera adusta* [16]	McIlvaine buffer with acetaminophen, bisphenol A, sulfamethoxazole and carbamazepine (20 mg/L) Mixture contained 250 μM of each compound.	25 °C, pH 6.0, 12 h 100% degradation of acetaminophen and bisphenol A; 20.5% degradation of carbamazepine; 22.0% and 19% degradation of sulfamethoxazole in presence of acetaminophen and other compounds, correspondently
Laccase from *T. versicolor* [24]	Milli-Q water with diclofenac, trimethoprim, carbamazepine and sulfamethoxazole; total concentration of PCPs was 1.25 or 5 mg/L in mixture.	25 °C, pH 6.8–6.9, 48 h, 80 rpm With single PCP: 100%, 95.0%, 82.0% and 56.0% degradation of diclofenac, trimethoprim, carbamazepine and sulfamethoxazole, correspondently With PCPs in mixture: 100%, 39.0%, 34.0% and 49.0% of same PCPs, correspondently
Cu^2+^-assisted laccase from *T. versicolor* [25]	Phosphate buffered saline with triclosan (10 μM)	25 °C, pH 6.0, 4 h, 3.0 mM Cu^2+^ 95.0% degradation of triclosan
Recombinant laccases from *Pleurotus ostreatus* [26]	Acetate buffer (50 mM) with sulfadiazine, sulfamethazine and sulfamethoxazole (100 mg/L)	25 °C, pH 4.8, 1 h 98.1%, 97.5%, and 97.8% degradation of sulfadiazine, sulfamethazine and sulfamethoxazole, correspondently
Soybean peroxidase [27]	Synthetic wastewater with triclosan, sulfamethoxazole, estrone, 17β-estradiol, 17α-ethynylestradiol, nonylphenol and octylphenol (5–50 mg/L)	0.05–0.5 mM H_2_O_2_, pH 6.0–7.0, 3 h 80.0% degradation of sulfamethoxazole and 95.0% degradation of all other PCPs
Laccase from *T. versicolor* and horseradish peroxidase [28]	Tap water and secondary wastewater with mixture of bisphenol A, 17α-ethinylestradiol, diclofenac and triclosan (10 mg/L of each compound) supplemented by 2.5% (*v*/*v*) McIlvaine’s buffer	25 °C, 20 h, 1% H_2_O_2_ pH 3.5–4.5 and 6.5–8.0 for peroxidase and laccase, correspondently; In tap water: 44.0, 68.0, 42.0 and 61.0% degradation of bisphenol A, 17α-ethinylestradiol, diclofenac and triclosan, was with laccase, correspondently; 83.0, 75.0, 49.0, and 56.0% degradation of same PCPs was with peroxidase; In wastewater: 81.0, 93.0, 38.0 and 72.0% degradation of same PCPs was with laccase; 63.0, 78.0, 17.0 and 54.0% degradation of same PCPs was with peroxidase

**Table 2:** Immobilized enzymes used for degradation of PCPs in various types of WW.

Biocatalyst [Reference]	Pollutant Concentration	Optimal Conditions of Enzymatic Action; Pollutant Degradation Efficiency
Laccase-graphene composite [29]	Phosphate buffer with labetalol (10 μM)	pH 7.0, 20 μM ABTS, 20 min 100% degradation of labetalol
Laccase from *Pycnoporus sanguineus* immobilized on TiO_2_ nanoparticles [30]	Wastewater from treatment plant with triclosan (1000 μg/L)	pH 7.9, 6 h, 93.0% electrooxidation/biocatalytic conversion
Laccase from *Tramates versicolor* immobilized as cross-linked enzyme aggregates [31]	Municipal wastewater with acetaminophen mefenamic acid, ketoprofen, fenofibrate, bezafibrate, indomethacin, trimethoprim, ibuprofen and ofloxacin (10–50 μg/L)	30 °C, pH 7.0, 6 h Degradation: 100.0% acetaminophen, 31.7% ofloxacin 65.1%, mefenamic acid 46.3%, ketoprofen, 90.0% fenofibrate 79.2%bezafibrate, 87.4% indomethacin 20.6% ibuprofen, 97.4% trimethoprim
Laccase from *T. versicolor* immobilized on laminated poly(acrylic acid) nanofibers [32]	Wastewater containing mixture of bisphenol A, 17α-ethinylestradiol, triclosan and diclofenac (10 mg/L)	20–22 °C, pH 7.0, 200 rpm 85.0%, 85.0%, 92.0% and 62.0% degradation of bisphenol A, 17α-ethinylestradiol, triclosan and diclofenac, correspondently
Laccase from *T. versicolor* immobilized on polyimide aerogel [33]	Secondary effluent from wastewater treatment plant with carbamazepine (20 ng/mL)	20 °C, 200 rpm, 24 h 74.0% degradation of carbamazepine
Laccase immobilized on/into poly(l-lactic acid)-co-poly(ε-caprolactone) (PLCL) electrospun nanofibers [34]	Acetate buffer solution with naproxen and diclofenac (1 mg/L)	25 °C, pH 3–5, 24 h 90.0–100% degradation of both PCPs
Native laccase from *T. versicolor* or immobilized by cross-linking using aminosilane magnetic nanoparticles [35]	Acetate buffer with diclofenac (1–20 μg/L)	22 °C, pH 5.0, 24 h Degradation of different concentrations of diclofenac under the action of native enzyme: 98.0% of 1 μg/L, 92.0% of 10 μg/L and 44.0% of 20 μg/L; Degradation of different concentrations of diclofenac under the action of immobilized enzyme: 90.0% of 1 μg/L, 53.0% of 10 μg/L and 26.0% of 20 μg/L
Laccase from *P. sanguineus* immobilized on TiO_2_ nanoparticles [36]	Phosphate/citric acid buffer or Groundwater samples with acetaminophen and diclofenac (10 mg/L)	25 °C, pH 4.0, 2–8 h 68.0% and 33.0% degradation of diclofenac for 8 h in the buffer and groundwater, correspondently; 90.0% and 84.0% degradation of acetaminophen for 2 h and 4 h in buffer and groundwater, correspondently
Laccase from *Phoma* sp. immobilized by cross-linking with poly(vinylidene fluoride) membrane [37]	Influent from a municipal wastewater treatment plant with a mixture of acetaminophen, bezafibrate, indometacin, ketoprofen, mefenamic acid and naproxen (10 μM each compound)	pH 7.0, 24 h Degradation: acetaminophen and mefenamic acid—85.0%, indometacin—11%, naproxen 16.0%, ketoprofen—15.0%, bezafibrate—12.0%
Laccase from *T. versicolor* immobilized on bentonite [38]	Citrate-phosphate buffer with tetracycline (10 mg/L)	30 °C, pH 5.0, 3 h 60.0% degradation of tetracycline
Laccase from *T. versicolor* immobilized on pinewood, pig manure or almond shell biochar [39]	Wastewater collected from urban community water treatment plant with diclofenac (500 µg/L)	25 °C, pH 6.4, 6 h 99.0% degradation of diclofenac
Laccase from *Myceliophthora thermophila* and *Pleurotus eryngii* immobilized on stevensite and holm oak biochar [40]	Sodium acetate buffer with oxytetracycline hydrochloride, tetracycline hydrochloride and chlortetracycline hydrochloride, sulfanilamide, sulfadiazine, sulfathiazole, sulfapyridine, sulfamethazine and sulfamethoxazole (0.1 mmol/L)	40 °C, pH 5.0, 24 h, 0.2 mmol/L ABTS; 84.0–100% and 11.0–64.0% degradation of tetracyxline antibiotics with laccase from *M. thermophile* and *P. eryngii*, respectively; 30.0–100% and 40.0–100.0% degradation of sulfonamide antibiotics with laccase from *M. thermophile* and *P. eryngii*, correspondently
Laccase from *T. hirsute* immobilized on poly(vinylidene fluoride) membrane modified with multi-walled carbon nanotubes [41]	Phosphate buffer with carbamazepine and diclofenac (5 mg/L)	25 °C, pH 5.0 27% degradation of carbamazepine for 48 h, 95% degradation of diclofenac for 4 h
Laccase from *T. versicolor* immobilized on onto the polyamide/polyethylenimine mat [42]	Wastewater with mixture of bisphenol A, 17α-ethinylestradiol, triclosan and diclofenac (10 g/L)	55 °C, pH 7.0, 3 µL H_2_O_2_, 72 h 18.0%, 33.0%, 64.0 and 7.0% degradation of bisphenol A, 17α-ethinylestradiol, triclosan and diclofenac, correspondently
Laccase from *Myceliophthora thermophila* immobilized on polypropylene beads [43]	50 mM Sodium phosphate buffer with morphine (1–60 g/L)	pH 7.0; 3 h; 100% degradation of morphine
Insolubilized tyrosinase and laccase from *T. versicolor* [11]	Municipal wastewater with naproxen, mefenamic acid, ibuprofen, ketoprofen, indomethacin, trimethoprim, ciprofloxacin, ofloxacin, caffeine, carbamazepine, bezafibrate, fenofibrate, atenolol (each 10 μg/L) and acetaminophen (35.5 μg/L)	20 °C, pH 7.5, 5 days; 100.0% degradation of 14 pollutants
β-Lactamase immobilized on the surface of *Aspergillus niger* (*A. niger*-Bla) [44]	Pharmaceutical wastewater with mixture of cefamezin (50 mg/L), amoxicillin and ampicillin (each 100 mg/L)	25–35 °C, pH 4–7, 20 days; 92.1%, 92.8% and 72.3% degradation of amoxicillin, ampicillin and cefamezin, correspondently
β-Lactamase immobilized on Fe_3_O_4_ magnetic nanoparticles [45]	Synthetic wastewater containing penicillin G (5–50 mg/L)	27–37 °C, pH 5.0–8.0, 5 min; 100% degradation of penicillin G
Chloroperoxidase immobilized on mesoporous dendritic silica particles [46]	Wastewater containing levofloxacin and rifaximin (20–100 μg/mL)	30 min; 88.0% degradation of 20 μg/mL antibiotics; 80.3% and 80.2% degradation of 100 μg/mL levofloxacin and 100 μg/mL rifaximin, correspondently
Horseradish or lignin peroxidase immobilized into magnetic sol-gel [8]	Acetate buffer with carbamazepine (17.6 µg/mL), paracetamol (100 µg/mL) and diclofenac (50 µg/mL)	55 °C, pH 3.0, H_2_O_2_, 72 h 100%, 100% and 50% degradation of carbamazepine, diclofenac and paracetamol, correspondently

**Table 3:** Bacterial and fungal cells as biocatalysts for the treatment of WW containing PCPs.

Biocatalyst [Reference]	Pollutant Concentration	Optimal Parameters and Degradation Efficiency
Bacterial Strains		
*Bacillus subtilis* [47]	Municipal wastewater with sulfamethoxazole and sulfadimethoxine (500 mg/L)	28–32 °C, pH 6.2–7.6 * COD—400–500 mg/L, 10 days; 100% degradation of antibiotics
*Bacillus velezensis* [48]	Synthetic wastewater containing tetracycline (100 mg/L)	35 °C, pH 7.0, 200 rpm, 8 days 99.2% degradation of tetracycline
Recombinant *E. coli* 6#P [49]	LB medium with sulfadiazine, sulfamethazine and sulfamethoxazole (1.0 mg/L)	37 °C, 60 h 92.0%, 89.0%, and 88.0% degradation of sulfadiazine, sulfamethazine and sulfamethoxazole, correspondently
*Acinetobacter* sp. [50]	Mineral salt medium with sulfamethoxazole, sulfadiazine and sulfamethazine (30 mg/L)	25 °C, pH 7.0, 150 rpm, 10 h 98.8%, 17.5% and 20.5% degradation of sulfamethoxazol, sulfadiazine and sulfamethazine, correspondently
*Labrys portucalensis* [51]	Minimal salts medium with diclofenac (34 µM)	25 °C, 130 rpm, sodium acetate (5.9 mM), 25 days 100.0% degradation of diclofenac
**Fungal strains**		
*Pycnoporus sanguineus*, *Phanerochaete chrysosporium* [52]	Medium based on sodium acetate buffer with ciprofloxacin, norfloxacin and sulfamethoxazole (10 mg/L)	30 °C, pH 5.0, 160 rpm Results of action of *P. sanguineus* cells for 2 days: 98.5%, 96.4% and 100% degradation of ciprofloxacin, norfloxacin and sulfamethoxazole; Results of action of *P. chrysosporium* cells for 8 days: 64.5%, 73.2% and 63.3% degradation of same PCPs; Efficiency of co-culture action: 100% degradation of all PCPs for 4 days
*A. niger*, *Mucor circinelloides*, *Trichoderma longibrachiatum*, *T. polyzona*, *Rhizopus microsporus* [53]	Synthetic wastewater with carbamazepine, diclofenac and ibuprofen (1 mg/L)	20 °C, pH 3.5–6.0, 120 rpm, 24 h; 91.9%, 99.3%, and 97.7 degradation of carbamazepine, diclofenac and ibuprofen, correspondently
*Pleurotus ostreatus* [54]	Dextrose Broth with antidepressants: paroxetine, sertraline, fluoxetine and citalopram. Serotonin-noradrenaline reuptake inhibitors: clomipramine, venlafaxine and mianserin (0.1–2.5 μg/mL)	26 °C, pH 6.5, 96 h; Degradation efficiency of sertraline—92.8%; paroxetine—93.7%; clomipramine—98.4%; mianserin—94.0; fluoxetine—85.1; citalopram—50.0%; venlafaxine—22%.
*P. ostreatus* [55]	Wastewater collected from wastewater treatment plant with bisphenol A, estrone, 17β-estradiol, estriol, 17α-ethinylestradiol, triclosan and 4-n-nonylphenol (total—455 ng/L)	28 °C, pH 7.2–8.3, 24 h; 76.0% degradation of all investigated PCPs
*P. ostreatus* [56]	Urban wastewater with sulfamethoxazole (423 ng/L), sulfapyridine (72.5 ng/L), sulfamerazine (13.5 ng/L) and sulfamonomethoxine (21.9 ng/L)	25 °C, 24 h; 100%, 100%, 93% and 95% degradation of sulfamerazine, sulfamonomethoxine, sulfamethoxazole and sulfapyridine, correspondently
*Ganoderma* *lucidum* [57]	Hospital wastewater with metoprolol and metoprolol acid (2 μg/L)	25 °C, pH 4.5, aeration 0.8 L/min, 7 days; 33.0% and 64.0% degradation of metoprolol and metoprolol acid, correspondently
*T. versicolor* and *G. lucidum* [58]	Synthetic medium (on base of dimethylsuccinate buffer) with O-desmethylvenlafaxine and venlafaxine (5 mg/L)	25 °C, pH 4.5; 100.0% degradation of O-desmethylvenlafaxine for 3 days; 70.0% degradation of venlafaxine for 15 days

* COD—Chemical Oxygen Demand.

**Table 4:** Immobilized bacterial and fungal cells used for the treatment of WW containing pharmaceutical pollutants.

Biocatalyst [Reference]	Pollutant Concentration	Optimal Parameters and Degradation Efficiency
Immobilized bacteria		
*Lactobacillus fermentum* LA6 immobilized into Ca-alginate gel with glutathione transferase activity [59]	Wastewater with oxytetracycline (200 mg/L)	30 °C, pH 7.5, 150 rpm, 0.03% H_2_O_2_, 0.04% SDS, 24 h; 89.1% degradation of oxytetracycline
*Bacillus thuringiensis* immobilized on loofah sponge [60]	Synthetic wastewater with naproxen (1 mg/L)	21–23 °C, pH 7.6, 15 days 90% degradation of naproxen
*Planococcus* sp. immobilized on loofah sponge [61]	Mineral salts medium with naproxen (15 mg/L)	25 °C, 130 rpm, 53 days; 100% degradation of naproxen
*Pseudomonas aeruginosa* immobilized on rice straw biochar [62]	Mineral salts medium with acenaphthene (3.5 mg/L)	37 °C, pH 7.0, 10 mg/L Triton X-100, 24 h; 78% degradation of acenaphthene
*P. putida* immobilized on Fe_3_O_4_/biochar composite [63]	Industrial pharmaceutical wastewater with calconcarboxylic acid (2.5 g/L)	20–22 °C, pH 7.0, 50 h; 84.0% removal efficiency
*P. stutzeri* immobilized on mesoporous silica nanoparticles [64]	Salt medium with alprazolam (100 mg/L)	22 °C, pH 7.4, 120 rpm, 20 days; 96% degradation of alprazolam
**Immobilized fungi**		
*Trihoderma versicolor* immobilized on rice husks [65]	Synthetic wastewater with azithromycin, sulfamethoxazole, trimethoprim, ciprofloxacin, florfenicol, lincomycin, ceftiofur hydrochloride, lorazepam, ketoprofen, acetaminophen, atenolol, albendazole, sertraline, sildenafil citrate, diphenhydramine, mefenamic acid and fluoxetine (1 mg/L) or real hospital wastewater	25 °C, pH 4.5, 336 h, 200 rpm, 3 L/min aeration; 60.0–100.0% degradation of PCPs occurred in synthetic wastewater, and 77.0%, 97.4%, 79.0%, 58.0%, 60.0% and 22.0% degradation of acetaminophen, gemfibrozil, caffeine, diphenhydramine, ibuprofen and carbamazepine occurred in non-sterile hospital wastewater, correspondently.
*T. versicolor* immobilized on rotating biological contactor (stainless steel disks for cells attachment) [66]	Urban wastewater with antipyrine, clofibric acid, atenolol, caffeine, carbamazepine, diclofenac, gemfibrozil, hydrochlorothiazide, ibuprofen, ranitidine, sulfamethoxazole and sulpiride (50 μg/L)	69,0%, 58.0, 88.0, 88.0, 61.0, 56.0, 66.0, 42.0, 95.0, 70.0, 87.0 and 95.0% degradation of antipyrine, clofibric acid, atenolol, caffeine, carbamazepine, diclofenac, gemfibrozil, hydrochlorothiazide, ibuprofen, ranitidine, sulfamethoxazole and sulpiride, correspondingly
*T. versicolor* immobilized on rotating biological contactors (propylene discs were covered by wooden pine sheets) [67]	Hospital wastewater with: antibiotics (amoxicillin, azithromycin, metronidazole, sulfamethoxazole, psychiatric drugs (carbamazepine, sulpiride and caffeine, β-blockers (atenolol, and metoprolol, nonsteroidal), anti-inflammatory drugs (diclofenac, and ibuprofen, IBP), analgesic (4-acetamidoantipyrine), cytotoxic (cyclophosphamide), contrast agent (iohexol), lipid regulator (gemfibrozil), chemical diuretic (hydrochlorothiazide), steroid hormone (progesterone), H_2_ histamine receptor antagonist (ranitidine) and endocrine disruptor (bisphenol A). The total concentration of PCPs was 16.32–16.51 mg/ L.	11–22 °C, pH 5.0–7.5, 75 days 99.9%, 87.0,% 97.8% and 98.5% degradation of cyclophosphamide, atenolol, azithromycin and sulpiride, 92.0% and 56.0% degradation of amoxicillin and progesterone, 0–5.0% degradation of 4-acetamidoantipyrine, iohexol and hydrochlorothiaziden, correspondingly. Other compounds were degraded by 40–80%

**Table 5:** Immobilized microalgae used for treatment of WW containing pharmaceutical pollutants.

Biocatalyst [Reference]	Pollutant Concentration	Conditions and Removal Efficiency
*Nannochloropsis* sp. immobilized into Ca-poly(vinyl alcohol) gel [76]	Medium f/2 with paracetamol ibuprofen and olanzapine (50 μg/mL)	25 °C, pH 6.0–8.0, 24 h photoperiod—16:8; removal efficiency of paracetamol—12.1%; ibuprofen—12.1%; olanzapine—40.0%
*Desmodesmus* sp. immobilized into Ca-alginate gel [77]	Domestic wastewater with 17β-estradiol (1 mg/L)	22 °C, pH 7.5, 72 h; removal efficiency—99.0%
*Chlorella vulgaris* immobilized into Ca-alginate/poly(vinyl alcohol) gel [78]	BG-11 medium with carbamazepine (80 mg/L)	25 °C, 12 days removal efficiency—87.0%
*C. vulgaris* immobilized into Ca-alginate gel with Al_2_O_3_ nanoparticles [79]	BG-11 medium with carbamazepine (100 mg/L)	28 °C, 4 days; removal efficiency—89.6%

**Table 6:** Microbial consortia used for the treatment of WW containing pharmaceutical pollutants.

Biocatalyst [Reference]	Pollutant Concentration	Conditions and Degradation Efficiency
Consortia in the Form of Suspensions		
Adapted consortium from anaerobic sludge [83]	Naproxen (1.2 mM)	35 °C, 11 days; 100% degradation of naproxen
Anaerobic sludge [84]	Ciprofloxacin (0.5–4.7 mg/L)	35 °C, 11–93 days; 20.0–76.4% degradation of ciprofloxacin
Bacterial consortium (adapted estuarine sediment and activated sludge) [85]	Paroxetine or Bezafibrate (1 mg/L)	500 mg/L of sodium acetate, dark conditions, 21 °C, pH 7, static or agitation conditions (130 rpm), 2 weeks; 97% degradation of both PCPs
Adapted consortium from anaerobic sludge [86]	Levofloxacin (10–50 mg/L)	20 mM glucose, 10 mM sulfate, 35 °C, dark conditions, 10 days; 39.6% degradation of levofloxacin
Nature microalgae-bacteria consortium containing *Chlorella sorokiniana* (80% of the total identified eukaryotic rRNA) and *Brevundimonas basaltis* (25% of the total identified prokaryotic rRNA) [81]	Cephalexin 50 μg/L	22 °C, 120 rpm, pH 8.0, 7 days 96.54% degradation of cephalexin
*Chlorella vulgaris, Scenedesmus obliquus* and algal–bacterial consortium present in the lagoon water [87]	Lagoon water from the effluent of Omemee wastewater Lagoon with mixed ibuprofen (50 μg/L), gemfibrozil (10 μg/L), triclosan (10 μg/L) and carbamazepine (10 μg/L)	22 °C, 11 days; >60% degradation of ibuprofen and triclodsan; gemfibrozil and carbamazepine were not decomposable
*C. vulgaris*, *Pseudonabaena acicularis*, *Scenedesmus acutus*, and activated sludge [88]	Model urban wastewater with ibuprofen (8.9 μg/L), naproxen (4.2 μg/L), salicylic acid (62 μg/L), triclosan (0.5 μg/L) and propylparaben (0.4 μg/L)	pH 7.7, 10 days; anaerobic-anoxic-aerobic photobioreactor; 94%, 52%, 98%, 100%, and 100% degradation of ibuprofen, naproxen, salicylic acid, triclosan and propylparaben, respectively
Green algae, diatom and cyanobacteria assemblages [89]	Mixed ibuprofen (45.7–100.4 μg/L), oxybenzone (6.9–14.1 μg/L), triclosan (2.9–3.4 μg/L), bisphenol A (2.2–3.9 μg/L) and N,N-diethyl-3-methylbenzamide (63.9–135.1 μg/L)	25 °C, 4 weeks, algal biofilm reactor; 70–100% degradation of each component
Adapted consortium (main cultures *Methylobacter*, *Pseudomonas*, and *Dokdonella* spp.) [90]	Ibuprofen (50 mg/L)	pH 7.0, 12 h 100% degradation of ibuprofen
**Immobilized consortia**		
Microbial consortium (*Alcaligenes faecalis, Staphylococcus haemolyticus*, *Staphyloccus aureus* and *Proteus mirabilis*) immobilized on *Luffa* [91]	Pharmaceutical wastewater of manufacturing industry COD—3530.1 mg/L; total Phenol—1580.2 mg/L; nitrates—5.2 mg/L; chlorides—87.1 mg/L; sulfates—32.3 mg/L; total suspended solids—286.9 mg/L	30–35 °C, pH 6.5–7.0, Batch fermentation for 36 h: dilution of medium by 4 times, the pollutants presented in wastewater were totally degraded into nontoxic compounds. Continuous treatment in aerobic fixed-film bioreactor for 61 days: organic loading rate—0.6–3.4 kg COD/m^3^/d. Average reduction of COD-96.8%, phenolic compounds-92.6% and of suspended solid—95.2%
*Phragmites australis* and bacterial consortium (*Acinetobacter lwoffii*, *Bacillus pumilus* and *Mesorihizobium* sp.) immobilized on polystyrene sheet [92]	Water contaminated by ciprofloxacin (100 mg/L)	Natural environmental conditions, 20 days 97.0% removal efficiency
The synthetic consortium (*Xenorhabdus* spp., *Pantoea agglomerans* and *Bacillus licheniformi*) immobilized in biofilm [93]	Oxaliplatin (780 mg/L)	20–22 °C, 21 days; bed biofilm reactor 94.0% degradation of oxaliplatin
Immobilized co-culture (*Klebsiella pneumoniae* CH3 and *Bacillus amyloliquefaciens* CS1) [94]	Wastewater with chlortetracycline (175 mg/L)	Polymer beads, pH 7.5, 10 days; 99.2% degradation of chlortetracycline
Activated sludge immobilized on glass porous beads (the predominant strains *Sphingomonas* and *Novosphingobium* sp.) [95]	Synthetic wastewater with ibuprofen (400–600 mg/L)	pH 7.0, aeration 550 mL/min, 24 h feeding periods, 160 days; 97.7% of degradation of ibuprofen
*C. vulgaris* immobilized Ca-alginate gel with powdered activated carbon and anaerobic bacterium consortium [96]	Anaerobically digested centrate with sulfamethoxazole (500 μg/L)	7 days; removal efficiency—99.0%

**Table 7:** Variants of hybrid physical–chemical–biocatalytic treatments of the PCPs.

Antibiotic [Reference]	Physical or/and Chemical Treatment	* BC/Co-Substrate	Removal Efficiency (%)
Levosulpiride (800 mg/L) [104]	Ozone (5.2 g/h) or activated carbon (2 g/L), 37 °C, pH 7.0, 150 rpm	*Alcaligenes faecalis* and *Exiguobacterium aurantiacum*	61.0% or 76.0% for 72 h in combination with ozone or activated carbon, correspondently
Metronidazole (10 mg/L) [105]	Two-chambered BES	Anaerobic activated sludge/glucose (1 g/L)	85% for 24 h
Nitrofurazone (NFZ) (50 mg/L) [106]	Dual-chamber BES with bio-cathode	NFZ-reducing consortium/ glucose 0.6 g/L	70% for 1 h
Cefazolin sodium (100 mg/L) [107]	Single-chamber BES with microbial bio-anode and activated carbon air-cathode	Aerobic activated sludge from the waste treatment plant brewery/NaAc (1.6 g/L), yeast extract (0.05 g/L)	70% for 31 h
Cefuroxime (0.5 mg/L) [108]	Two-chamber BES	Activated sludge/ Glucose (1 g/L)	90% for 12 h
Sulfamethoxazole or Tetracycline (each 200 mg/L) [109]	Three-dimensional biofilm-electrode BES reactor	Anaerobic sludge from municipal wastewater treatment plant/ glucose (225 mg/L)	72–94% and 83–96% of sulfamethoxazole and tetracycline for 40 h, correspondently
Chloramphenicol (30 mg/L) [110]	Two-chamber BES	Anaerobic sludge	84% for 48 h
Cefradine (300 mg/L) [111]	AOP: H_2_O_2_/Fe(II)-based Fenton reactions	Algae *Chlorella pyrenoidosa*	85% for 48 h
Amoxicillin and cefradine [112]	UV-irradiation at 365 nm	Algae *Scenedesmus obliquus*	100% for 24 h
Sulfamethoxazole or norfloxacin (each 32 mg/L) [113]	Hybrid BES with Fenton reactions (γ-FeOOH GPCA air-cathode)	Anaerobic sludge from the secondary sedimentation basin of wastewater treatment plant	96–97% for 40 h
Metronidazole (80 mg/L) [114]	Hybrid two-chamber BES with combining the catalytic photo-Fenton and an electro-Fenton (FeIII) processes on Mo/W coated graphite felt cathodes	Anaerobic sludge/ sodium acetate (1.0 g/L)	95–97% for 0.5–1 h
Ofloxacin (36–145 mg/L) [115]	Hybrid BES with LiNbO_3_/CF photocatalytic cathode	PANi@CNTs/SS bioanode/ Glucose (0.7 g/L)	70–87% for 7 h
Wastewater from the bulk pharmaceutical manufacturing [116]	Fenton reactions with FeSO_4_ × 7H_2_O and H_2_O_2_	Aerobic activated sludge from pharmaceutical manufacturing	Removing of 53.8% COD for 2 h

* AOP—advanced oxidation processes, BC—biocatalyst, BES—bioelectrochemical system, GPCA—grapheme polyacrylamide carbonized aerogel, NFZ—nitrofurazone, PANi@CNTs/SS—polyaniline@carbon nanotubes/stainless steel.

## Data Availability

Not applicable.
